# Exploring the Antimicrobial and Pharmacological Potential of NF22 as a Potent Inhibitor of *E. coli* DNA Gyrase: An In Vitro and In Silico Study

**DOI:** 10.3390/pharmaceutics14122768

**Published:** 2022-12-10

**Authors:** Samman Munir, Mohsin Khurshid, Matloob Ahmad, Usman Ali Ashfaq, Magdi E. A. Zaki

**Affiliations:** 1 Department of Bioinformatics and Biotechnology, Government College University Faisalabad, Faisalabad 38000, Pakistan; 2 Department of Microbiology, Government College University Faisalabad, Faisalabad 38000, Pakistan; 3 Department of Chemistry, Government College University, Faisalabad 38000, Pakistan; 4 Department of Chemistry, Faculty of Science, Imam Mohammad Ibn Saud Islamic University (IMSIU), Riyadh 11623, Saudi Arabia

**Keywords:** quinolones, norfloxacin, *E. coli*, DNA gyrase, antimicrobial agents

## Abstract

Toward the search for novel antimicrobial agents to control pathogenic *E. coli*-associated infections, a series of novel norfloxacin derivatives were screened for antimicrobial activities. The norfloxacin derivative, 1-ethyl-6-fluoro-7-(4-(2-(2-(3-hydroxybenzylidene)hydrazinyl)-2-oxoethyl)piperazin-1-yl)-4-oxo-1,4-dihydroquinoline-3-carboxylic acid (NF22) demonstrated excellent antibacterial activities against *E. coli* ATCC 25922 (MIC = 0.0625 μg/mL) and MDR *E. coli* 1–3 (MIC = 1, 2 and 1 µg/mL). The time-kill kinetic studies have demonstrated that the NF22 was advantageous over norfloxacin and ciprofloxacin in killing the control and MDR *E. coli* strains. The checkerboard assay showed that NF22 in combination with tetracycline had a synergistic effect against the *E. coli* strains. The experimental findings are supported by molecular modeling studies on DNA gyrase, explaining the interactions involved for compound NF22, compared to norfloxacin and ciprofloxacin. Further, the compound was also evaluated for various pharmacokinetics (absorption, metabolism, distribution, toxicity and excretion) as well as drug-likeness properties. Our data have highlighted the potential of norfloxacin by restoring its efficacy against *E. coli* which could lead to the development of new antimicrobial agents.

## 1. Introduction

The use of antibacterial agents within clinical settings was one of the most significant scientific accomplishments of the 20th century [1]. The development of antimicrobials undoubtedly improved animal and human health considerably. However, antimicrobial resistance (AMR) has become one of the critical public health threats across the globe [2,3]. The data from the past few years have suggested that the infection caused by MDR pathogens is increasing even in technically advanced countries, which is leading to an increased number of deaths [4]. The inappropriate and extensive utilization is considered the leading cause of resistance among the bacteria that is represented by multi-drug resistant (MDR) phenotypes [5].

Escherichia coli (*E. coli*) is one of the most common facultative anaerobic, gram-negative bacteria that cause many hospitals and community-acquired clinically important infections and represents a leading cause of death among people of all ages. In recent decades, the resistance of *E. coli* strains towards antimicrobial agents has grown steadily, and because of the increasing resistance combined with its array of infections, it has now been included in the priority pathogens list of the World Health Organization (WHO), along with other members of the family Enterobacteriaceae [6].

Fluoroquinolone (FQ) is a family of antibiotics that have been extensively utilized for over half a century to manage various bacterial infections [7]. FQs primarily target type-II topoisomerase: topoisomerase IV (Topo IV) and gyrase (Gyr) and act by inhibiting DNA supercoiling in bacteria, preventing the replication of bacterial DNA and eventually leading to cell death [8]. Generally, these enzymes are present in both Gram-negative as well as Gram-positive pathogens and depending upon the host organism along with the quinolone utilized for the management, either of these enzymes can serve as a key target of a particular drug [9]. In Gram-negative organisms, the main target of FQs is DNA Gyr [10]. Gyr is composed of two A subunits (GyrA) and two B subunits (GyrB), creating a heterotetrameric architecture (A2B2) that produces an active enzyme. The GyrA subunit is responsible for non-catalytic interactions with DNA, while the GyrB effectively catalysis the DNA strands cutting and rejoining [11].

In the past few years, AMR against FQs is rising internationally, particularly in *E. coli,* causing numerous infections [12]. According to an international surveillance report, almost thirty percent of all *E. coli* isolates are FQ-resistant, limiting empirical therapy with these antibacterial agents [13].

The demand for the continuous development of novel antimicrobials has become too high, having considered this significant threat, to keep up with the bacterial adaptation. Certain FQs possess potential therapeutic applications, addressing a large number of pathologies, for example, bacterial infections, malaria, cancer, viral infections, neurodegenerative diseases, fungal infections, tuberculosis and immunodepression. Furthermore, the physiochemical properties of the FQs, specifically their structure and reactivity, have also triggered interest, maintaining long-term attention towards this antibiotic class for many decades [14]. In addition, the FQ moiety also acts as a scaffold for developing novel bioactive molecules, and studies with FQ derivatives have revealed that this class possesses broad-spectrum biological activities, like anticancer and antimicrobial activities, etc. [15].

**Figure 1:**
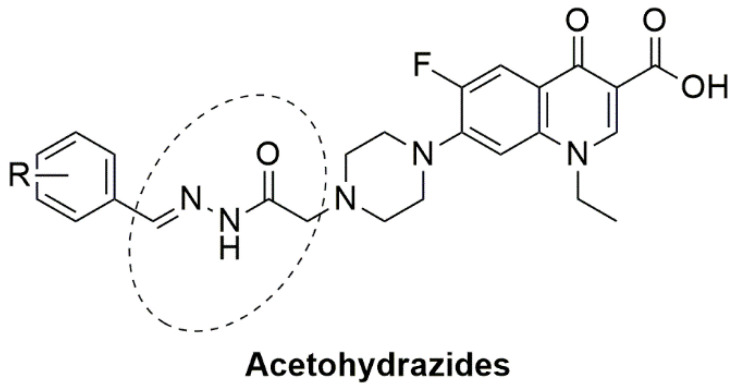
The general chemical structures of norfloxacin-based acetohydrazides [19,20].

On the other hand, schiff bases (SB) are widely known for their various biological and pharmacological activities, like, antifungal, anti-inflammatory, antiviral and antibacterial activities [16,17,18]. Therefore, based on the above-mentioned facts, the present study focused primarily on the evaluation of the antimicrobial potential of synthetic NOR analogs against *E. coli*, supported by multiple pharmacological and computational studies, for assessing the true antimicrobial potential of the most effective compound as a lead molecule for drug development. The titled compound NF-22 was selected from a series of synthesized norfloxacin-based acetohydrazides and acetamides (Figure 1) [19,20]. These compounds were screened against various strains of *E. coli* and among them, the most effective compound NF-22 was selected for further investigations.

## 2. Materials and Methods

### 2.1. In Vitro Study

#### 2.1.1. Materials and Instrument

The glassware used in the present research work was made by Pyrex ^®^. The antibiotics (Ciprofloxacin and Norfloxacin) and culture media were purchased from Oxoid, (Hampshire, UK). All the in vitro experiments were performed in a biosafety cabinet that was disinfected with an appropriate disinfectant before and after each experiment. The optical density of the bacterial suspension was measured at 450 nm with a Microplate Reader (BioTek, ELx808, GEN5). The *E. coli* ATCC 25922 was provided by Mr. Muhammad Sohail, Chughtai Lab, Lahore, Pakistan. The MDR *E. coli* were the laboratory stocks at the Department of Microbiology Government College University Faisalabad, Pakistan that were the clinical isolates that were identified by using the VITEK 2 system (bioMérieux, France). The MIC to multiple drugs was evaluated by the broth microdilution method [21]. 

#### 2.1.2. Norfloxacin Derivative Library

**Figure 2:**
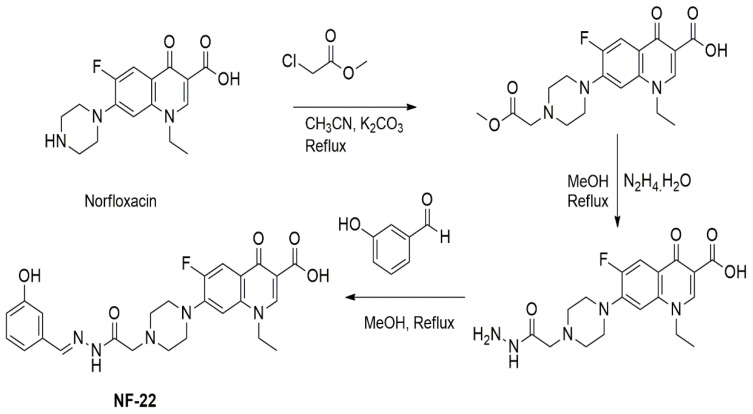
Schematic outline for synthesis of 1-ethyl-6-fluoro-7-(4-(2-(2-(3-hydroxybenzylidene)hydrazinyl)-2-oxoethyl)piperazin-1-yl)-4-oxo-1,4-dihydroquinoline-3-carboxylic acid analogue.

The synthesis, as well as characterization of the NOR derivatives screened in the present study, have already been reported [19,20]. A schematic outline of 1-ethyl-6-fluoro-7-(4-(2-(2-(3-hydroxybenzylidene)hydrazinyl)-2-oxoethyl)piperazin-1-yl)-4-oxo-1,4-dihydroquinoline-3-carboxylic acid (NF22) synthesis is detailed in Figure 2.

### 2.2. In Vitro Antibacterial Activity

MIC, the lowest concentration of the study compound that prevents visible bacterial growth, was determined for each compound through the broth microdilution (MD) technique as per the guidelines of CLSI (Clinical Laboratory Standards Institute) using cation-adjusted Mueller Hinton (MH) broth. The single bacterial colony was cultured for 2–4 h at 37 °C to yield approximately 10^8^ colony forming units per ml (CFU/mL) of bacterial cells. The microbial cultures were further diluted to obtain 10^6^ CFU/mL into MHB. A solution of all the study compounds was prepared in DMSO at a concentration of 51,200 µg/mL, which were further diluted to 512 µg/mL. After that, 100 µL of the twofold diluted compounds (512, 256, 128, 64, 32, 16, 8, 4, 2, 1, 0.5, 0.125, 0.0625 and 0.03125 µg/mL) was added within each well of a 96 well microtiter plate containing 100 µL of the 4X MH broth, followed by 100 µL of the bacterial suspension. The antimicrobial ciprofloxacin (CIP) and norfloxacin (NOR) were utilized as positive controls and DMSO as a negative control. The microtiter plates were kept in the incubator for 18–24 h at 37 °C. The tests were carried out in triplicate, and the reading was observed at 625 nm using a spectrophotometer.

#### 2.2.1. Time Kill Assay

The single *E. coli* colony was cultured in 1 mL of fresh MH broth overnight. The suspension was further diluted with MH broth and kept in a shaking incubator at 37 °C for 2–3 h to get 10^6^ CFU/mL. After that, the microbial suspension was separated into 5 different glass tubes incubated with various concentrations of the analog NF22 (0.03125 µg/mL and 0.0625 µg/mL) and standard drugs NOR (0.5 µg/mL) and CIP (0.25 µg/mL) and one tube as growth control for 0, 2, 4, 6, 8, 10 and 12 h correspondingly. At each particular time, 1 mL of the sample was removed and serial 10-fold dilutions were made in saline. The viable bacterial cells were then determined using the plate count method which involved plating 10 µL of dilution onto a nutrient agar plate. The plates were kept in the incubator for 24 h at 37 °C. Data were analyzed by plotting log10 CFU/mL versus time (hrs.). Bacteriostatic or bactericidal effects were, correspondingly, defined as <3 log10 or ≥3 log10 decreases in cell count compared to the initial bacterial inoculum [22].

#### 2.2.2. Synergy Testing

The checkerboard test was carried out similarly to the MIC assay in 96-well plates. Each 96-well plate comprised serial dilutions of NF22 analog and different drugs (tetracycline (TET) and ampicillin (AMP)) in a checkerboard manner as described earlier [23]. The initial concentrations of the study compound and conventional antibiotics were those used previously in the determination of MIC. In total, 7 dilutions of NF22 and 11 dilutions of AMP and TET were examined. The microtiter plate was incubated for 24 h at 37 °C. During each test, a growth control comprising the medium only (neither drug nor NF22 analog) was also included. The fractional inhibitory concentration (FIC) index was computed using the equation:

$$\Sigma \mathrm{FIC}=\frac{\mathrm{MIC}\;\mathrm{drug}\;A\;\left(\mathrm{combination}\right)}{\mathrm{MIC}\;\mathrm{drug}\;A\;\left(\mathrm{alone}\right)}+\frac{\mathrm{MIC}\;\mathrm{drug}\;B\;\left(\mathrm{combination}\right)}{\mathrm{MIC}\;\mathrm{drug}\;B\;\left(\mathrm{alone}\right)}$$

The FIC index value of less than 0.5 denoted synergism, the FIC index value between 0.5–4 denoted additive/indifference effect, while the FIC index value of greater than 4 denoted as antagonism.

#### 2.2.3. Cytotoxicity Assay

The cytotoxicity of compound NF22 was evaluated using the Vero cell line (ATCC, Manassas, VA, USA) through MTS assay. Briefly, Vero cells were initially seeded in a 96-well plate at the cell density of 2 × 10^4^ cells per well and then allowed to incubate in a CO_2_ incubator (5% CO_2_) at 37 °C for 24 h. After that, cells were treated with various concentrations of NF22 analog (100 µM–0.78 µM) for 24 h. Control cells containing 0.1% DMSO were also concurrently employed. After the incubation, the culture medium comprising the study compound was replaced with a fresh medium (100 µL/well). Then, 20 µL MTS solution was added into each well and kept in incubator for 3 h. The quantity of formazan produced was measured at 490 nm using a spectrophotometer [24]. The percentage viability with respect to DMSO-treated cells was determined using the following formula:

$$\mathrm{Percentage}\;\mathrm{viability}=\frac{\mathrm{Test}\;570\;\mathrm{nm}-630\;\mathrm{nm}\;}{C\mathrm{ontrol}\;570\;\mathrm{nm}-630\;\mathrm{nm}}\;\times \;100$$

### 2.3. In Silico Analysis

#### 2.3.1. Structure Prediction

The biological activity of proteins is determined by their overall three-dimensional (3D) structure. Changes in the structure of proteins may alter the function of the protein and unfortunately cause the deadliest disease. NOR agents are reported to particularly inhibit the subunit A of Gyr enzyme, a type 2 topoisomerase (Topo), which seems to be vital for bacterial DNA replication [25]. No high-resolution crystal structure is available for Gyr holoenzyme (A_2_B_2_), nevertheless, numerous crystal structures are present for separate domains from different bacterial species [26,27]. The X-ray crystal structure of *E. coli* Gyr subunit A (GyrA) was not available in Protein Data Bank (PDB). Highly accurate protein structure predictions by deep neural networks such as AlphaFold2 have a tremendous impact on structural biology and beyond [28]. Initially, the sequence of DNA GyrA was downloaded from Uniprot using uniport id: Q8XE30. The sequence was then subjected to Alphfold2 for structure prediction. AlphaFold2 is a neural network-based protein structure prediction program created by Google DeepMind. The AlphaFold2-based prediction was run with the “single sequence” mode. The algorithm first searches for homologous sequences with existing structures to use as a scaffold on which to place the new sequence. Additionally, we also specified that the algorithm should run an Amber relaxation procedure to repair any structural violations in the predicted model. A list of models was generated by AlphaFold2, but top-ranked model was selected on the basis of highest overall pLDDT scores. pLDDT is the per-residue estimate of its confidence on a scale from 0–100.

#### 2.3.2. Structure Evaluation

Accurate assessment of the 3D protein model is considered a key element of structure prediction. Recently, it has been seen as an outbreak of emerging sequencing technologies that enable researchers to make ground-breaking discoveries in the domain of computational structure biology. The development of highly effective and rapidly approved methods for structure assessment has paved novel ways to qualitatively predict protein structures. In the present work, the predicted protein structure was further qualitatively estimated using 3 independent programs: Verify 3D, ERRAT, and Ramachandran plot analysis [29,30]. The Chimera and PyRx software were used for initial quality estimation, and energy minimization of the predicted structure [31]. Further, the CASTp tool was employed to find out atoms of residues that constitute the active site surface for the target protein [32].

#### 2.3.3. Structure Activity Relationship (SAR) and Molecular Docking

SAR analysis was performed using the Datawarrior drug discovery tool. DataWarrior is free, open-source software that combines both cutting-edge and traditional chemoinformatics techniques in a single environment for data processing and structural activity relationship of the specific compounds [33]. Furthermore, the interaction between *E. coli* GyrA and compound NF22 was validated through a molecular docking approach. Molecular docking lies at the heart of drug discovery process as it helps in understanding the binding affinity that exists between compounds and their related targets. In the framework of the current study, Autodock Vina of PyRx, a computational software, was utilized to perform docking among target protein and active compound NF22. The docked pose with the lowest value of binding energy (BE) and root mean square deviation (RMSD) was selected for further study. For the docked complex, the model exhibiting the highest absolute value of BE was considered accurate. Moreover, Chimera X and discovery studio were used for the visualization of interaction among active compound and target protein [34].

### 2.4. In Silico ADME/T Study

The compound NF22 was subjected to an in silico ADME/T profiling through pkCSM and SwissADME tools [35,36]. The structural properties of the compound were predicted based on Lipinski’s RO5 [37]. ADME/T properties like water solubility, gastrointestinal (GI) absorption, the permeability of the blood–brain barrier (BBB), lipophilicity, and CYP450 inhibition were forecasted.

## 3. Results

### 3.1. Antibacterial Activity

The antimicrobial potential of target compounds was evaluated in vitro using standard methods against gram-negative bacteria *E. coli* (ATCC 25922) and three MDR *E. coli* (*E. coli* 1, *E. coli* 2 and *E. coli* 3). The results were expressed as the MIC and then compared with the standard drugs CIP and NOR (Table 1).

Among the compounds tested, NF22 exhibited potent antimicrobial activity against *E. coli*, ATCC 25922 (MIC = 0.0625 μg/mL) which was 2–4 fold more effective compared to positive control CIP (MIC = 0.125 µg/mL) and NOR (MIC = 0.25 µg/mL). Moreover, the compound NF22 also showed the strong antibacterial effect against clinical *E. coli* 1–3 (MIC = 1, 2 and 1 µg/mL) which was several times effective than CIP (MIC = 8–32 µg/mL) and NOR (MIC = 32–64 µg/mL).

### 3.2. Time-Kill Curves

In the current study, only compound NF22 was selected for time-kill analysis as it exhibited greater activity against *E. coli* ATCC 25922 (MIC = 0.0625 µg/mL) and *E. coli* 1–3 (MIC = 1–2 µg/mL).

**Figure 3:**
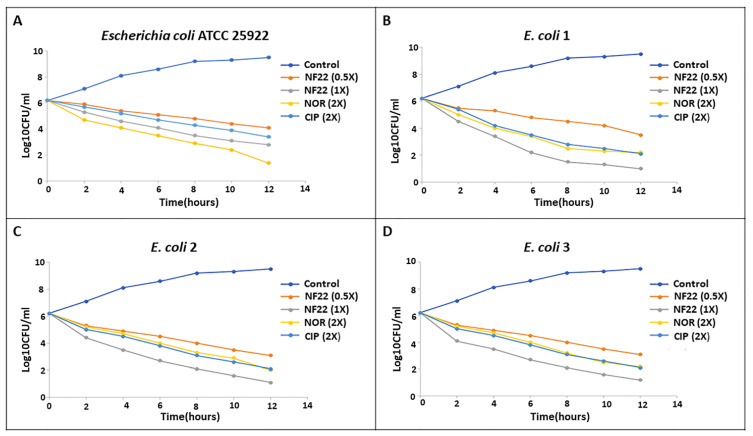
Time kill curves for NF22 against pathogens. (**A**) *E. coli* were grown to exponential phase and experimental groups include analog NF22 (at 0.03125 and 0.0625 µg/mL), NOR (at 0.5 µg/mL), CIP (at 0.25 µg/mL) and control group (sterile water); (**B**) *E. coli* 1 were grown to exponential phase and exposed to compound NF22 (at 0.03125 and 0.0625 µg/mL), NOR (at 0.5 µg/mL), CIP (at 0.25 µg/mL) and control group (sterile water); (**C**) *E. coli* 2 were grown to exponential phase and exposed to compound NF22 (at 0.03125 and 0.0625 µg/mL), NOR (at 0.5 µg/mL), CIP (at 0.25 µg/mL) and control group (sterile water); (**D**) *E. coli* 3 were grown to exponential phase and exposed to compound NF22 (at 0.03125 and 0.0625 µg/mL), NOR (at 0.5 µg/mL), CIP (at 0.25 µg/mL) and control group (sterile water). Each experiment was conducted three times.

Time-kill analyses revealed that the potent compound NF22 has a bactericidal effect. As illustrated in Figure 3, the analogue eliminated more than 99.9% bacteria by 12 h against susceptible and MDR *E. coli*. At 1 × MIC concentration, the compound NF22 exhibited relatively faster bactericidal activity against MDR *E. coli* in comparison to NOR (2X) and CIP (2X). Therefore, the findings suggest that the compound NF22 has the advantage over widely used antimicrobials CIP and NOR in killing pathogenic *E. coli* strains.

### 3.3. Synergistic Activity of NF22 with Other Antimicrobials

The synergistic effect of NF22 in combination with tetracycline (TET) and ampicillin (AMP) on microbial growth was determined through the checkerboard test (Table 2). The combination of NF22 and TET gave a synergistic effect against *E. coli* 1–3, whereas the interaction between CIP and TET as well as NOR and TET was indifferent. In the case of AMP, indifferent effects were observed when combined with NOR, TET and NF22 against *E. coli* 1–3.

### 3.4. Cytotoxicity Analysis of NF22

**Figure 4:**
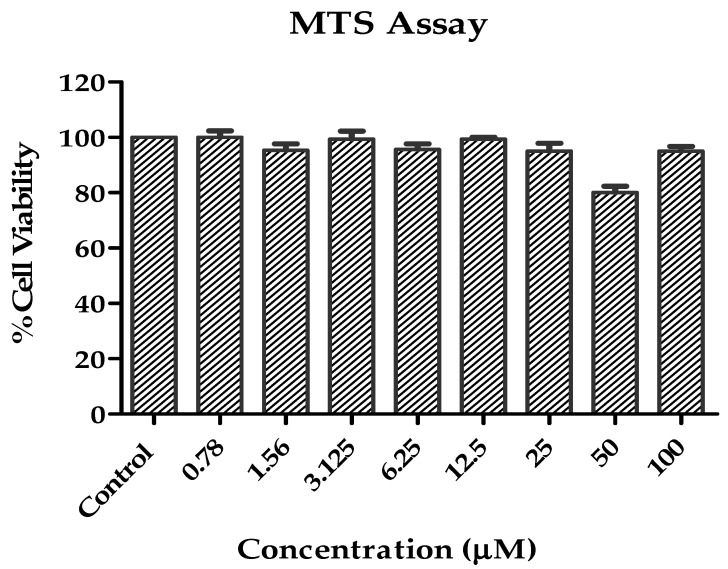
Response of NF22 compound on Vero cell line (normal).

The cytotoxicity of the NF22 was evaluated using a colorimetric MTS assay. Vero cells were treated for 24 h with various concentrations of compound ranging from 100 to 0.78 µM. The study compound was found to not exhibit any cellular toxicity up to 100 µM concentrations (Figure 4).

### 3.5. In Silico Analysis

#### 3.5.1. Structure Analysis

A 3D structural model of the GyrA was generated using Alphafold2. In its validation at the 14th edition of the Critical Assessment of protein Structure Prediction (CASP14), the predictions generated were demonstrated to be comparative to experimental structures [38]. The AlphaFold2-based prediction was run with the “mmseqs2” mode by ColabFold. Furthermore, to further improve the quality of protein structure, we also used amber force fields to fix structural violations in the 3D structure of nucleoproteins. Later, AlphaFold2 generated five models for each input sequence. In all five models predicted by AlphaFold2, the top-ranked model was selected based on the pLDDT values.

The evaluation of the predicted model of the Gyr protein revealed that 88.6% of residues were present in favored regions, 7.9% residues within allowed regions, 2.6% within the generously allowed region, and 0.9% in the disallowed region. Conclusively, the assessment of 3D protein model validated that approximately 90% residues were present within the favored as well as allowed regions, thereby confirming the high quality of the predicted model. VERIFY 3D tool predicted that 84.88% of the residues possessed an average 3D-1D score of more than 0.2, which further validates the model within the context of sequence/structure compatibility. ERRAT, called a quality factor, exhibited that the quality score of the Gyr model was 95.5556. The greater the score, the better the overall quality of the predicted 3D model. Altogether, the findings verify that the predicted model is of high-quality.

#### 3.5.2. Structure Activity Relationship and Molecular Docking Analysis

SAR analysis was performed using the datawarrior automatic SAR analysis drug discovery tool. Different conformations of NF22 compound were generated using the Random, low energy biased algorithm and MMMFF94s force-filed and the best conformation was selected for the molecular docking with 197 kcal/mol energy. Further SAR analysis of NF22 based on MMFF94 forcefield revealed the compound efficiency (0.25474 nmol/L), lipophilic ligand efficiency (6.1036 nmol/L), ligand efficiency lipophilic price (2.2815 nmol), molecular flexibility (0.45875 nmol/L) and molecular complexity (0.90826 nmol/L). All these values showed the effectiveness and liability of the compound. Some drug-likeness properties of NF22 also revealed the reliability of this compound compared to others. Properties such as clogP (0.5812), clogS (−4.065), total surface area (364.97), polar surface area (125.78) and ultimately drug-likeness were also found to be effective. This analysis revealed the effectiveness of NF22 and it was further carried for molecular docking.

Molecular docking was carried out to analyze the binding interactions of NF22 with the GyrA enzyme and the findings were then compared with the standard drugs (CIP and NOR). The estimated binding affinity towards the active site of gyrase and the resultant RMSD value of the target compound NF22 and standard drugs are shown in Table 3. The study compound revealed a binding affinity of −13.4 kcal/mol and 1.786 RMSD value with *E. coli* GyrA enzyme. The molecular-docking analysis demonstrated that the study compound showed stronger binding energies with the target protein compared to standard drugs.

**Figure 5:**
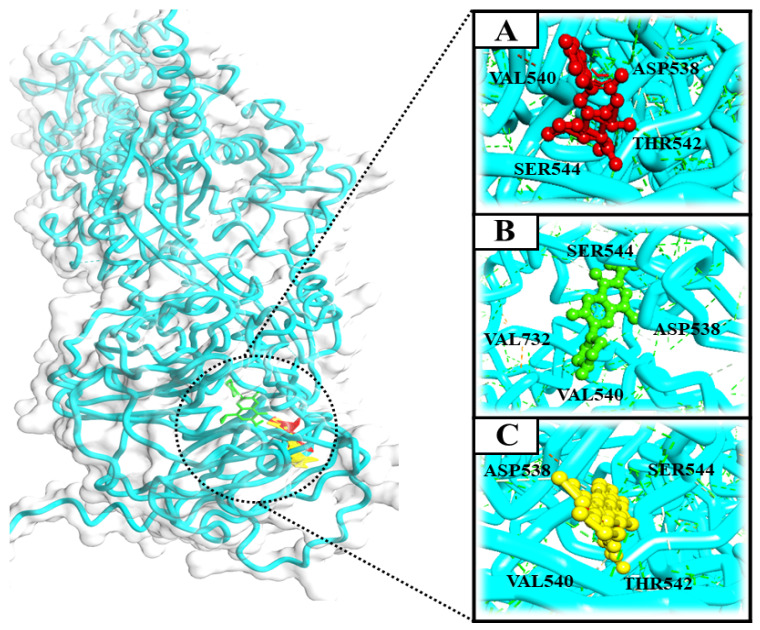
Docked complexes of standard drugs (**A**) NOR and (**B**) CIP and (**C**) analog NF22 with *E. coli* DNA gyrase A.

Docking studies revealed that the compound NF-22 maintained a similar mode of interaction predicted for NOR, with important interactions with Ser 544, Thr 542, Asp 538 and Val 540 from *E. coli* gyrase. While in the case of CIP, the target compound exhibited a similar interaction with amino-acid residue Ser 544, Val 540 and Asp 538 (Figure 5).

### 3.6. ADME/T Study

For the ADME/T study, computational tools have been considered a suitable alternative to laborious experimental methods, particularly in the initial phases [39]. The compound’s physicochemical characteristics were computationally examined to assess the drug-like characteristics based on RO5 (rule of 5): no. Of hydrogen-bond acceptors 10, MW 500, MiLogP 5, and no. of hydrogen-bond donors 5 (Table 4).

Moreover, various PK parameters were evaluated to estimate the distribution, elimination, absorption, toxicity, and metabolism of the NF22 (Table 5). The compound was found to be greatly absorbed within GI tract, not cross the BBB, and not an inhibitor of CYP enzymes, indicating that the target compound could be considered a promising clinical drug candidate.

## 4. Discussion

Microbial resistance towards antibiotics has become an international health challenge with profound consequences. *E. coli* is considered the best indicator of AMR in microbial communities as it is recognized as an important reservoir of antibiotic-resistant genes [40]. Moreover, *E. coli* resistance towards FQs is of great concern since the prevalence of resistance mechanisms through horizontal gene transfer has increased considerably in the last few decades. Moreover, the discovery of antimicrobials is under consistent challenge, resulting in a small amount of novel antibacterial agents being introduced into hospital settings. Hence, possible approaches are urgently required to enhance the number of treatment options.

The compounds were tested for their antimicrobial efficacy against Gram-negative organism *E. coli* ATCC 25922 and three MDR bacteria *E. coli* 1–3. gram. While considering the MIC, the compound NF22 exhibited the most potent antibacterial activity against *E. coli* ATCC 25922 (MIC = 0.0625 µg/mL) compared to NOR (MIC = 0.25 µg/mL) and CIP (MIC = 0.125 µg/mL). The antimicrobial potential of the study compounds against three MDR *E. coli* was further evaluated. It was noteworthy that the compound NF22 also exhibited a strong antibacterial effect against *E. coli* 1–3 (MIC = 1, 2 and 1 µg/mL, correspondingly), which was multiple fold more effective than the standard drugs CIP (MIC = 8–32 µg/mL) and NOR (MIC = 32–64 µg/mL).

Time-kill kinetics tests have been used extensively for in-vitro analyses of novel antibacterial agents because these give qualitative (descriptive) data regarding the pharmacodynamic characteristics of antibacterial agents [41]. The assay was performed with compound NF22 only as it displayed the highest MIC value against *E. coli* ATCC 25922 and MDR strains of *E. coli*. The results revealed that the compound NF22 exhibited a similar reduction in bacterial colonies (*E. coli* 25922) at 1X MIC concentration compared to that of CIP (2X) after 12 h of incubation, however showed a bit less effectiveness compared to the NOR at 2X concentration. The analog NF22 displayed better bactericidal activity at 1X concentration towards MDR *E. coli* compared to the CIP and NOR. The finding is comparable to that displayed by NF derivative 5K, which revealed superior bactericidal activity against methicillin-resistant *S. aureus* than for *S. aureus* [42].

The use of antimicrobial agents in combination can enhance their therapeutic effects because of the different interactions of each drug. Different drugs might have distinct targets and affect each target site to obtain the response that results in increased biological effects within the cells. Conversely, different drugs can influence a similar target, which might lead to an agonistic effect [43]. In addition, combining antibacterial agents has been effectively applied to improve the bactericidal effect, optimize dosing regimens, reduce the emergence of antimicrobial resistance and prevent any treatment failure [44]. In the present study, the combination of NF22 and TET was detected to have a synergistic effect against *E. coli* 1–3. Numerous investigations have shown the antagonistic effects between inhibitors of protein synthesis and DNA synthesis inhibitors [45,46]. Nevertheless, a recent investigation revealed synergy among tigecycline (bacteriostatic) and enrofloxacin (bactericidal) drugs against TET-resistant *E. coli* [47]. The synergism between two antimicrobial agents might be because of the improved uptake ability of one drug via other drugs; synergistic interaction based on improved uptake has been demonstrated in previous investigations [48]. Our findings are similar to the previous study that showed that the synergy between the combination of nalidixic acid (quinolone) and tetracycline against the multidrug-resistant (MDR) *E. coli* and *A. baumannii* clinical isolates but not against the susceptible isolates [49].

Moreover, both NOR and AMP are bactericidal drugs; however, they act at different target sites and via different mechanisms on microbial cells. AMP is involved in inhibiting bacterial cell wall integrity and formation whereas NOR targets bacterial gyrase [50]. The indifference effect detected among NOR and AMP, CIP, and AMP as well as NF22 and AMP might be because of the negative impact caused by the complex formation of two compounds.

Cellular toxicity of the NF22 was also evaluated against Vero cells using MTS assay. This assay has frequently been utilized to examine the cellular toxicity of study compounds [51,52]. The findings exhibited that the NF22 has no cytotoxic effect on Vero cell line (normal epithelial cells).

The usefulness of bacterial Gyr as a potential target of antimicrobial agents arises from its mechanism of DNA supercoiling [53]. The complete details of the mechanism are under investigation; however, a model, generally called as “two-gate model”, has strongly been supported by structural as well as biochemical data [54]. The most widely studied Gyr is isolated from *E. coli*, which possess two subunits (Gyr A and Gyr B) of molecular mass 97 and 90 kDa, correspondingly [54]. The FQs generally, and NOR particularly, are bactericidal agents. These drugs are found to particularly inhibit the subunit A of the bacterial DNA gyrase enzyme which is critical for the replication of DNA. Nevertheless, the precise mechanism through which the FQs cause death of bacterial cells has yet to be validated [25,55]. The study compound NF22 and standard drugs, CIP and NOR, were docked within the catalytic region of GyrA. An increased interaction was detected with the target protein, as can be observed by the increase of binding affinity in the case of NF22 as compared to CIP and NOR. Moreover, it was observed that the compound NF22 maintained a similar mode of action predicted for NOR, with important interactions with Asp 538, Val 540, Thr 542 and Ser 544. Compared to CIP, analogue NF22 displayed similar interactions with amino-acid residues Asp 538, Val 540 and Ser 544.

The ADME/T study plays a vital role in understanding the pharmacokinetic characteristics of the compounds for medical treatment. Drug likeness analyses determine the possibility of a compound serving as a drug regarding its bioavailability [56]. The estimation of these characteristics is quite necessary for the process of drug development and a poor ADME/T profile often fails novel chemical substances during clinical studies [57]. The findings of pkCSM and swissADME provided valuable data about the target compound. The compound was estimated not to pass through the BBB, be highly absorbed in the GI tract, and not modified in drug biotransformation, suggesting that the compound can be an ideal candidate for drug development.

## 5. Conclusions

Here we have presented the antimicrobial potential of a norfloxacin derivative against the reference and MDR strains of *E. coli*. The antibacterial assays have exhibited that NF22 has excellent activities against *E. coli*. The docking of NF22 within the catalytic region of gyrase has shown an increased binding affinity compared to ciprofloxacin and norfloxacin. The in-silico absorption, distribution, metabolism, excretion, and toxicity modeling for rational drug design using ADME/T-related prediction models could be helpful in the drug development process. Here, the findings have shown that the target compound was not able to pass the blood–brain barrier with good absorption from the GIT which suggests the NF22 as an ideal candidate for drug development. The results are suggestive of NF22 as an attractive drug with enhanced efficacy against *E. coli*. Further in vivo studies are suggested to validate the findings using animal models.

## Figures and Tables

**Table 1:** MIC (µg/mL) of norfloxacin derivatives (NF1–NF22) and standard drugs.

Compound	*E. coli* (ATCC 25922)	*E. coli* 1	*E. coli* 2	*E. coli* 3
NF1	8	256	128	64
NF2	16	128	64	128
NF3	16	64	64	256
NF4	32	128	256	32
NF5	64	256	32	64
NF6	16	32	128	64
NF7	32	32	64	256
NF18	8	256	64	256
NF19	8	128	32	32
NF20	16	64	256	64
NF21	32	64	64	16
NF22	0.0625	1	2	1
Norfloxacin	0.25	32	64	32
Ciprofloxacin	0.125	8	32	8

**Table 2:** Synergy evaluation of NF22 in combination with different antimicrobials.

Antibiotic Combination	Antibiotics	*E. coli* 1					*E. coli* 2					*E. coli* 3				
		MIC ^a^	MIC ^c^	FIC	FICI	Interaction	MIC ^a^	MIC ^c^	FIC	FICI	Interaction	MIC ^a^	MIC ^c^	FIC	FICI	Interaction
NOR-TET	NOR	32	16	0.5	0.75	Indifference	64	32	0.5	1	Indifference	32	16	0.5	1	Indifference
	TET	64	16	0.25			128	64	0.5			128	64	0.5		
CIP-TET	CIP	8	4	0.5	0.75	Indifference	32	16	0.5	1	Indifference	8	4	0.5	1	Indifference
	TET	64	16	0.25			128	64	0.5			128	64	0.5		
NF22-TET	NF22	1	0.125	0.125	0.375	Synergistic	2	0.5	0.25	0.5	Synergistic	1	0.25	0.25	0.5	Synergistic
	TET	64	16	0.25			128	32	0.25			128	32	0.25		
NOR-AMP	NOR	32	4	0.25	0.75	Indifference	64	32	2	2.5	Indifference	32	16	0.5	1	Indifference
	AMP	512	256	0.5			512	256	0.5			1024	512	0.5		
CIP-AMP	CIP	8	2	0.25	0.75	Indifference	32	16	0.5	1	Indifference	8	4	0.5	1	Indifference
	AMP	512	256	0.5			512	256	0.5			1024	512	0.5		
NF22-AMP	NF22	1	0.25	0.25	0.75	Indifference	2	1	0.5	1	Indifference	1	0.5	0.5	1	Indifference
	AMP	512	256	0.5			512	256	0.5			1024	512	0.5		

^a^ MIC: MIC of one drug alone. ^c^ MIC of drugs in combination.

**Table 3:** Docking results of compound NF22 against *E. coli* DNA gyrase A.

Sr. No.	Compound Name	Compound Structure	Binding Affinity (kcal/mol)	RMSD	Interacting Residues
1	CIP	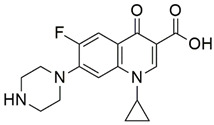	−6.6	2.884	Ser 544
					Val 732
					Asp 538
					Val 540
2	NOR	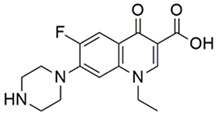	−8	2.537	Ser 544
					Thr 542
					Asp 538
					Val 540
3	NF22	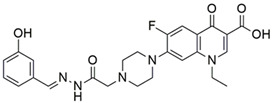	−13.4	1.786	Ser 544
					Asp 538
					Val 540
					Thr 542

**Table 4:** Results of NF22 evaluated for Lipinski rule.

Compound	Molecular Weight (g/mol) < 500	Number of HBA < 10	Number of HBD < 5	MLogP < 5
NF22	495.50	8	3	1.02

**Table 5:** ADMET properties enlisting Metabolism, Toxicity and Absorption related drug-likeness profiling of compound NF22.

Absorption and Metabolism								Distribution and Toxicity	
Blood–Brain Barrier	Gastrointestinal Absorption	P-glycoprotein substrate	CYP2C19 inhibitor	CYP2D6 inhibitor	CYP2C9 inhibitor	CYP3A4 inhibitor	CYP1A2 inhibitor	Subcellular localization	AMES Toxicity
No	Yes	Yes	No	No	No	No	No	Mitochondria	No

## Data Availability

Not applicable.
